# Understanding the Psychosocial Impact of Assistive Technologies for People With Visual Impairments: Protocol for a Scoping Review

**DOI:** 10.2196/65056

**Published:** 2025-02-13

**Authors:** Raul Szekely, Catherine Holloway, Maryam Bandukda

**Affiliations:** 1 Computer Science Department University College London London United Kingdom

**Keywords:** assistive technology, psychosocial impact, quality of life, visual impairment, scoping review protocol, mobile phone

## Abstract

**Background:**

There has been a rapid growth in the literature on the design and evaluation of assistive technologies for people with visual impairments; yet, there is a lack of a comprehensive analysis of the existing literature on the classification of immediate-, short-, medium-, and long-term psychosocial impact of assistive technologies on the quality of life of people with visual impairments.

**Objective:**

This protocol outlines the methodology for a scoping review aimed at identifying and synthesizing the existing literature on the psychosocial impact of assistive technologies on the quality of life of people with visual impairments.

**Methods:**

The review will include primary research studies published in English between 2019 and 2024 that focus on the psychosocial outcomes of assistive technologies for people with visual impairments. Eligible studies will involve participants with visual impairments, of all ages and across various settings, examining psychological (eg, emotional well-being and self-esteem) and social outcomes (eg, social participation and support). Searches will be conducted across 7 electronic research databases: CINAHL (EBSCO), PsycINFO (EBSCO), ACM Digital Library, IEEE Xplore, Scopus, Web of Science, and Google Scholar (first 100 records). Studies will undergo screening and selection based on predefined eligibility criteria, with data extraction focusing on publication details, study design, population characteristics, type of assistive technology, and psychosocial impacts. Results will be summarized using descriptive statistics, charts, and narrative synthesis.

**Results:**

The database search, conducted in July 2024, identified 1145 records, which will be screened and analyzed in subsequent stages of the review process. This protocol outlines the planned approach for identifying, categorizing, and synthesizing evidence. The study findings are anticipated to be finalized and submitted for publication in a peer-reviewed journal by February 2025.

**Conclusions:**

This study will synthesize the recent body of work on the psychosocial impact of assistive technologies for people with visual impairments and recommendations for researchers and designers interested in this research area.

**Trial Registration:**

Open Science Framework 10.17605/OSF.IO/SK7N8; https://osf.io/4gc5t

**International Registered Report Identifier (IRRID):**

DERR1-10.2196/65056

## Introduction

### Background

Globally, visual impairment affects approximately 1 billion people [1]. Visual impairment significantly impacts people’s quality of life, affecting activities of daily living, education, employment, and social interactions [2-4]. The most prominent effects of vision loss are loss of independence and social isolation, leading to anxiety, depression, and other mental health conditions [4]. Due to this, many people with visual impairments experience low self-esteem and self-efficacy in their mobility and social interaction [5]. Furthermore, the participation of people with visual impairments in leisure activities is low. Where they do, people with visual impairments participate in passive leisure activities (eg, watching television and listening to the radio) rather than actively participating in physical activities, social interaction, and sports in outdoor places [6]. Research shows that people with visual impairments have low mental health outcomes and overall quality of life compared to sighted people [4,7,8].

The World Health Organization [9] defines assistive technology (AT) as an umbrella term for assistive products, systems, and services designed to maintain or improve one’s functioning related to cognition, communication, hearing, mobility, self-care, and vision, therefore promoting health, well-being, inclusion, and participation. The European Parliament research report on AT for people with disabilities [10] distinguishes among five types of ATs for blindness and visual impairment: (1) haptic aids (eg, the white cane, the traditional Braille system, embossed pictures, advanced Braille apps, advanced canes, haptic aids for computer use, and matrices of point stimuli), (2) traveling aids (eg, low-technology haptic aids, obstacle and object location detectors, electronic travel devices, assistive apps in mobile phone technology, embedded technologies, and mixed systems), (3) AT for accessible information and communication, (4) AT for daily living (eg, labeling systems, talking readers, tactile and vibrating clocks and alarms, talking kitchen tools, and talking wallets and purses), and (5) phone and tablet apps (eg, magnification apps, color detection apps, money identification apps, object identification apps, scan and read apps, screen reading apps, voice recognition apps, location and GPS apps, and Braille apps).

Mashiata et al [11] classified ATs for visual impairment into four categories: (1) based on portability (eg, nonwearable devices such as smart canes and assistant robots and wearable travel aids such as head-mounted, ear aids, belt-mounted, blind shoes, glasses, and gloves), (2) based on navigation (eg, audio-tactile maps, indoor, indoor-outdoor, and outdoor such as smart city or urban navigation, vehicle detection, airport accessibility, shopping guide, and pedestrian navigation), (3) based on detection (eg, object recognition and obstacle detection, including vehicle detection, pedestrian detection, staircase, and daily life objects), and (4) based on smartphone assistance (eg, digital assistants, mobile apps, including voice maps, voice search, and mobile games).

Over recent decades, disability has stopped being viewed solely in functional terms. Frameworks such as the International Classification of Functioning, Disability and Health and the World Health Organization Quality of Life [12] now emphasize the importance of psychological and social dimensions in understanding and enhancing the quality of life for individuals with disabilities. However, much of the existing research on the impact of ATs has primarily focused on the functional outcomes associated with their use and attributed the poor uptake of these technologies to functional issues [13,14]. Two key points should be raised in relation to this. First, ATs continue to be designed from a biomedical, deficiency-oriented rather than a psychosocial, person-centered approach, failing to effectively fulfill users’ needs [15]. Second, researchers have overlooked the role of psychosocial factors in the perception and use of ATs, which may better explain their acceptance and uptake, prompting the development of instruments such as the Psychosocial Impact of Assistive Devices Scale [13] to address this gap.

As with other disabilities, prior research has mainly focused on the functional outcomes of AT and rehabilitation interventions for people with visual impairments [16-18]; yet, there is a lack of a comprehensive review of the literature to understand the psychological and social impact of AT for this population. This review aims, therefore, to comprehensively examine the research investigating the short-, medium-, and long-term psychosocial impact of AT for people with visual impairments.

### Objectives and Review Questions

This scoping review aims to answer the following questions: (1) What psychological and social outcomes are associated with the use of ATs among people with visual impairments? (2) What methods and instruments are used to measure the psychosocial impact and outcomes of ATs for people with visual impairments? (3) What are the key trends in the literature in relation to the population characteristics, countries of study, settings, type of ATs examined, impact period, and research methodologies used to assess the psychosocial impact of ATs for people with visual impairments?

## Methods

### Ethical Considerations

Ethics approval is not required for this study, as human participants were not involved.

### Study Design

This scoping review will be conducted following the framework proposed by Arksey and O’Malley [19], which includes six stages: (1) identifying the research question; (2) identifying relevant studies; (3) study selection; (4) charting the data; (5) collating, summarizing, and reporting the results; and (6) consultation. Methodological recommendations from the Joanna Briggs Institute [20] and the PRISMA-ScR (Preferred Reporting Items for Systematic Reviews and Meta-Analyses extension for Scoping Reviews) checklist [21] will also inform the process.

### Stage 1: Identifying the Research Question

The primary research question guiding this scoping review is: What are the psychosocial impacts of ATs on people with visual impairments? This question arose from the recognition that while ATs are often evaluated for their functional efficacy, their broader psychosocial impacts have been less explored, despite these impacts potentially being equally or more important in encouraging the uptake and long-term use of these technologies and improving quality of life among users. This scoping review draws from theoretical frameworks such as the World Health Organization Quality of Life framework [12] and Schalock and Alonso’s Quality of Life model [22] to conceptualize psychosocial impacts. That is, psychological outcomes refer to the impact of ATs on the mental and emotional state of the individual, including positive feelings (happiness and life satisfaction), negative feelings (anxiety, depression, and stress), and self-esteem. Social outcomes refer to the impact of ATs on the individual’s social interactions, support, and participation in community and societal activities.

### Stage 2: Identifying Relevant Studies

An initial limited search was conducted by the research team to inform the development and refinement of the search strategy. A university librarian was also consulted at this stage to help identify the databases and refine the search strategy. The search included variations and combinations of the following key concepts:

- AT (“assistive technol*” OR “adaptive technol*” OR “assistive aid*” OR “assistive equipment*” OR “assistive device*” OR “assistive product*” OR “assistive service*” OR “assistive interv*” OR “sensory aid*”).
- Visual impairment (“visual impair*” OR “vision impair*” OR “impaired vision” OR “sight impairment” OR “visual loss” OR “vision loss” OR “vision defect” OR “visual handicap” OR “blind” OR “blindness” OR “low vision” OR “partial* sight*” OR “partial vision” OR “visual* disorder*” OR “vision disord*” OR “visual* disab*” OR “vision disab*” OR “eye disord*”).

Next, a full literature search of peer-reviewed journal papers and conference proceedings was conducted across 7 electronic databases, including CINAHL (EBSCO) and PsycINFO (EBSCO), as well as ACM Digital Library, IEEE Xplore, Scopus, Web of Science, and Google Scholar (first 100 records). During the initial limited search, PubMed was considered as a potential database but eventually excluded, as its results heavily targeted biomedical and clinical aspects, which did not align with the focus on psychosocial impacts. Instead, CINAHL was selected for its broader coverage of health care topics and more holistic aspects of care, including well-being and quality of life.

To focus on the most up-to-date literature and to capture the latest developments in the field of ATs for visual impairments, the search was limited to studies published in the past 5 years (2019-2024). Furthermore, the search was limited to studies written in English only. A full literature search was conducted in July 2024, which produced 1942 results.

### Stage 3: Study Selection

A systematic approach will be used for study selection. Eligibility criteria have been developed to ensure the relevance and quality of included studies. From a *population* perspective, studies will be included if they focus on people with visual impairments, including children and adults, irrespective of the diagnosis and inclusion criteria used by individual studies. At a *concept* level, studies will be included if they (1) refer to the use of AT by people with visual impairments and (2) focus on the psychosocial impact or outcomes associated with the use of ATs. *Context-wise*, studies conducted in any country or setting, including health care, community, education, and work, and across all age groups will be considered. Primary research, including quantitative, qualitative, and mixed method studies reported in peer-reviewed journal papers or conference proceedings, will be included in this review. Conference abstracts only will be excluded. Secondary research (eg, literature reviews and meta-analyses) and nonempirical works (eg, theoretical papers, conceptual frameworks, opinion pieces, and editorials) may be consulted during the review process but will not be included. The review will only include research that includes the design or evaluation of an AT intervention focusing on impact.

**Figure 1:**
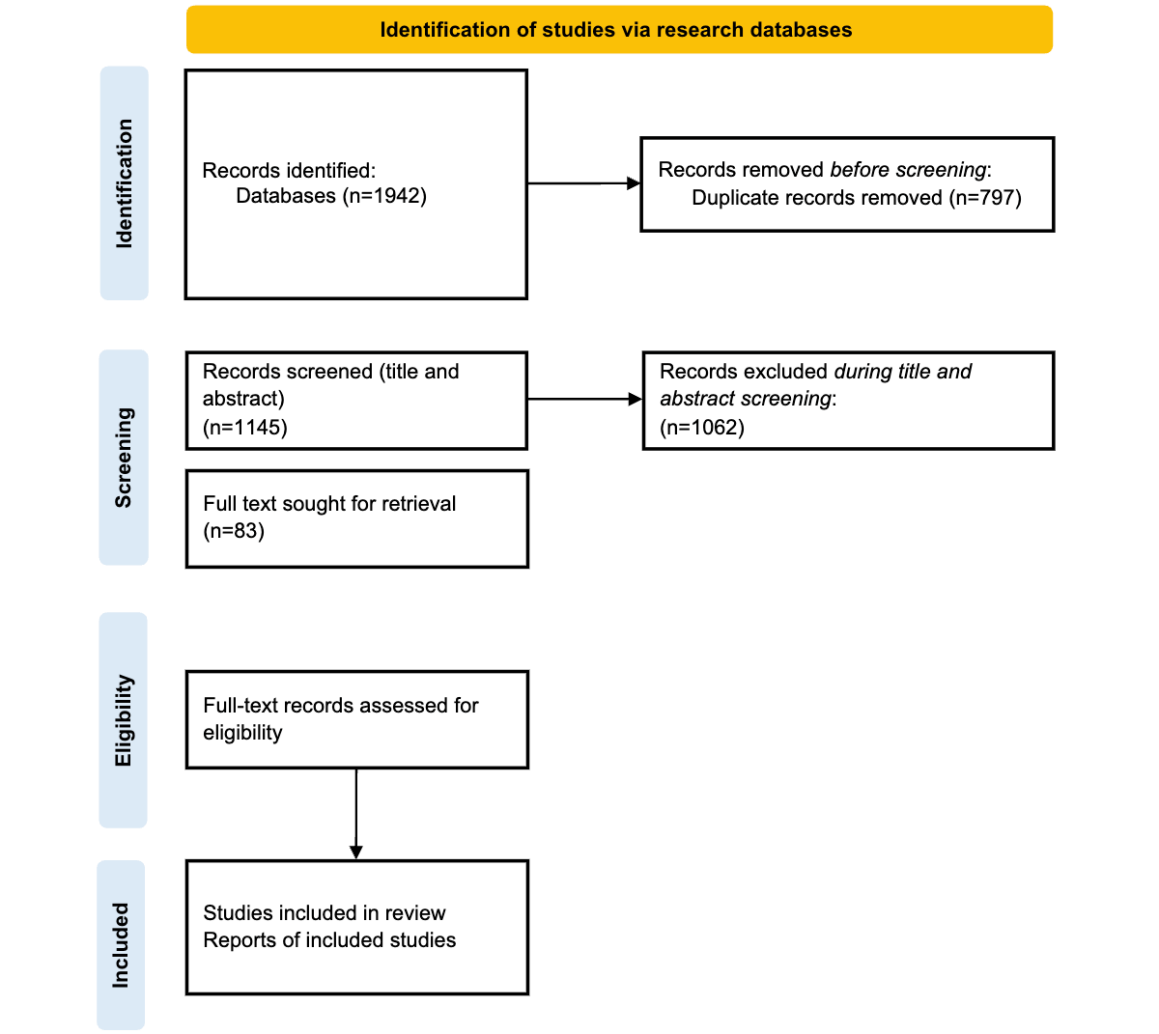
PRISMA-ScR (Preferred Reporting Items for Systematic Reviews and Meta-Analyses Extension for Scoping Reviews) flow diagram illustrating the study selection process.

Following the literature search, all retrieved studies will be collated and uploaded into a web-based literature review tool, Rayyan (Rayyan Systems Inc) [23], where duplicates will be removed. A random sample of 25 papers from the overall dataset will be first reviewed for pilot-testing of the source selector criteria. Following this, the reviewers will meet to discuss discrepancies and adapt the criteria based on the insights from the pilot test. Independently, 2 reviewers (RS and MB) will then conduct a screening of titles and abstracts to determine their potential eligibility for inclusion. The full texts of potentially eligible studies will be then retrieved and reviewed in detail for final inclusion. Reasons for excluding sources of evidence at the full-text stage that do not meet the eligibility criteria will be documented and reported in the scoping review. Any disagreements between reviewers (RS and MB) at each stage of the study selection process will be resolved through consensus or by consulting a third reviewer (CH). The search results and the study inclusion process will be reported in full in the final scoping review and presented in a PRISMA-ScR flow diagram (Figure 1 and Multimedia Appendix 1).

### Stage 4: Charting the Data

Data from the studies included in the scoping review will be extracted using a standardized table to ensure consistency and comprehensiveness. The data extraction table will be piloted on a subset of studies and will be revised as necessary. The information to be extracted from each study includes the authors, year of publication, title, source of publication, type of publication, country of study, aim of the study, population and sample characteristics, research design, type of AT or intervention, setting, outcomes, instruments or impact measures, key findings, and the impact period categorized as immediate (less than 1 month), short-term (1 to 6 months), medium-term (6 to 12 months), and long-term (more than 12 months). To ensure the accuracy of the process, a second reviewer (MB) will cross-check the data extracted from at least 25% (n=7) of all included studies. Any discrepancies between reviewers will be resolved through consensus or consultation with a third reviewer (CH).

### Stage 5: Collating, Summarizing, and Reporting Results

Descriptive statistics will be used to summarize the overall characteristics of the included studies. These will cover the number of studies, the distribution of studies by publication year, population characteristics, country of origin, study setting, type of ATs examined, impact period, and research methodologies used. Charts and diagrams will also be used to support data presentation. Tables will be constructed to synthesize the psychosocial outcomes reported in the included studies. Tables will detail the methods and instruments used to measure the psychosocial impacts of ATs, including information on specific tools or questionnaires, their reliability and validity, and the context of their app. Additionally, tables will summarize the main findings of the studies included in the review. Furthermore, a narrative summary will accompany the tabular or charted results, providing a description of the literature on the psychosocial impact of ATs on people with visual impairments, including key themes and trends observed. It will also identify gaps in the current body of knowledge, while recommendations for future research will be made based on the identified gaps or inconsistencies.

### Stage 6: Consultation

To expand the relevance and applicability of the findings arising from this scoping review, consultations will be conducted with stakeholders, including AT researchers, policy makers, and users. In particular, AT users will be provided with a summary of findings and invited to provide feedback through a written commentary. Their insights will inform the interpretation of results and the development of recommendations for practice and policy.

## Results

This scoping review protocol was submitted to the Open Science Framework [24] on July 17, 2024. The database search was conducted in July 2024, and 1942 records were identified. During identification, 797 records were identified as duplicates and removed. Next, the title and abstract screening was conducted for 1145, of which 1062 records were excluded. Finally, 83 records were included for full-text screening. Data extraction and synthesis, as well as paper preparation, are currently underway. The paper should be submitted in February 2025.

## Discussion

### Expected Findings

The findings from this scoping review will shed light on the psychosocial impacts of ATs for people with visual impairments. The review will also explore the methods and instruments used to measure these outcomes and will identify key trends in the literature. These findings are expected to inform the development of a global evidence database mapping the impact of AT for people with visual impairments, with a view to extending it to other disabilities and long-term conditions, including other sensory impairments, mental health conditions, neurodevelopmental conditions, intellectual disabilities, and physical disabilities. The database is also expected to serve as a resource for researchers, clinicians, AT developers, policy makers, and other stakeholders, providing accessible and up-to-date evidence on the impact of AT. It is intended to facilitate evidence-based decision-making, support the development of guidelines, interventions, and policies, as well as identify gaps in the current research landscape. Findings will be presented at relevant conferences and shared with stakeholders (eg, disability and health care organizations, AT developers, and policy makers).

### Strengths and Limitations

This scoping review is a comprehensive attempt to map the interdisciplinary literature on the psychosocial impact of AT for people with visual impairments. A full literature search of peer-reviewed journal papers and conference proceedings was conducted across 7 interdisciplinary electronic databases, including CINAHL (EBSCO) and PsycINFO (EBSCO) for literature from health care and psychology, as well as ACM Digital Library, IEEE Xplore for human-computer interaction, computing, and accessibility and AT-related literature, and Scopus, Web of Science, and Google Scholar (first 100 records) to include cross-disciplinary literature. The review will follow the PRISMA-ScR checklist that is specific for scoping reviews [21] to ensure a high level of quality and transparency. To this end, the scoping review protocol has been preregistered with the Open Science Framework [24].

One limitation of this scoping review is the exclusion of previous reviews and non–peer-reviewed publications. Additionally, the review only includes research published in English between 2019 and 2024. Due to the limited scope and time constraints of this review, a comprehensive quality assessment will not be conducted. Finally, this review does not include a full literature search on PubMed due to the clinical focus of search results from initial searches.

## Appendix

Multimedia Appendix 1 PRISMA-Scr (Preferred Reporting Items for Systematic Reviews and Meta-Analyses Extension for Scoping Reviews) checklist.

## Abbreviations

AT: assistive technology
PRISMA-ScR: Preferred Reporting Items for Systematic Reviews and Meta-Analyses extension for Scoping Reviews

## Data Availability

The datasets generated or analyzed during this study are available from the corresponding author on reasonable request.
